# Investigating Olfactory Gene Variation and Odour Identification in Older Adults

**DOI:** 10.3390/genes12050669

**Published:** 2021-04-29

**Authors:** Siddharth Raj, Anbupalam Thalamuthu, Nicola J Armstrong, Margaret J Wright, John B Kwok, Julian N Trollor, David Ames, Peter R Schofield, Henry Brodaty, Perminder S Sachdev, Karen A Mather

**Affiliations:** 1 Centre for Healthy Brain Ageing, Faculty of Medicine, School of Psychiatry, University of New South Wales (UNSW), Sydney, NSW 2031, Australia; siddharth.raj@gmail.com (S.R.); a.thalamuthu@unsw.edu.au (A.T.); J.Trollor@unsw.edu.au (J.N.T.); h.brodaty@unsw.edu.au (H.B.); p.sachdev@unsw.edu.au (P.S.S.); 2 Department of Mathematics and Statistics, Curtin University, Perth, WA 6102, Australia; nicola.armstrong@curtin.edu.au; 3 Queensland Brain Institute, University of Queensland, St. Lucia, QLD 4072, Australia; margie.wright@uq.edu.au; 4 Centre for Advanced Imaging, University of Queensland, St. Lucia, QLD 4072, Australia; 5 School of Medical Sciences, University of Sydney, Sydney, NSW 2006, Australia; john.kwok@sydney.edu.au; 6 Department of Developmental Disability Neuropsychiatry, UNSW, Sydney, NSW 2031, Australia; 7 National Ageing Research Institute, Parkville, VIC 3052, Australia; dames@unimelb.edu.au; 8 Academic Unit for Psychiatry of Old Age, University of Melbourne, St George’s Hospital, Kew, Melbourne, VIC 3010, Australia; 9 Neuroscience Research Australia, Sydney, NSW 2031, Australia; p.schofield@neura.edu.au; 10 School of Medical Sciences, UNSW, Sydney, NSW 2031, Australia; 11 Dementia Collaborative Research Centre Assessment and Better Care, UNSW, Sydney, NSW 2031, Australia; 12 Neuropsychiatric Institute, the Prince of Wales Hospital, Sydney, NSW 2031, Australia

**Keywords:** olfaction, odour identification, genetics, ageing

## Abstract

Ageing is associated with a decrease in odour identification. Additionally, deficits in olfaction have been linked to age-related disease and mortality. Heritability studies suggest genetic variation contributes to olfactory identification. The olfactory receptor (OR) gene family is the largest in the human genome and responsible for overall odour identification. In this study, we sought to find olfactory gene family variants associated with individual and overall odour identification and to examine the relationships between polygenic risk scores (PRS) for olfactory-related phenotypes and olfaction. Participants were Caucasian older adults from the Sydney Memory and Ageing Study and the Older Australian Twins Study with genome-wide genotyping data (*n* = 1395, mean age = 75.52 ± 6.45). The Brief-Smell Identification Test (BSIT) was administered in both cohorts. PRS were calculated from independent GWAS summary statistics for Alzheimer’s disease (AD), white matter hyperintensities (WMH), Parkinson’s disease (PD), hippocampal volume and smoking. Associations with olfactory receptor genes (*n* = 967), previously identified candidate olfaction-related SNPs (*n* = 36) and different PRS with BSIT scores (total and individual smells) were examined. All of the relationships were analysed using generalised linear mixed models (GLMM), adjusted for age and sex. Genes with suggestive evidence for odour identification were found for 8 of the 12 BSIT items. Thirteen out of 36 candidate SNPs previously identified from the literature were suggestively associated with several individual BSIT items but not total score. PRS for smoking, WMH and PD were negatively associated with chocolate identification. This is the first study to conduct genetic analyses with individual odorant identification, which found suggestive olfactory-related genes and genetic variants for multiple individual BSIT odours. Replication in independent and larger cohorts is needed.

## 1. Introduction

Olfaction is a trait that is important for nutrition, well-being and quality of life. In the general population, the prevalence of olfactory impairment is low (3.8–5.8%) [1]. However, ageing is associated with a decline in the ability to smell with more than 75% of individuals over 80 years old exhibiting a major olfactory deficit [2]. Impaired olfactory ability has been linked to hippocampal atrophy, high white matter hyperintensity (WMH) volumes and increased amyloid burden [1,3]. It has also been associated with the incidence of neurodegenerative diseases, including Parkinson’s disease (PD), Alzheimer’s disease (AD), global cognitive performance in older adults [1,3,4] and increased risk of mortality [5]. Age-related olfactory dysfunction may be due to degeneration of the olfactory bulb and epithelium [6], ossification of olfactory foramina [7] deterioration of the transduction properties of olfactory receptor neurons [8] and/or neuropathological changes due to neurodegenerative disease [9] and head trauma. Long term exposure to chemicals, for example via smoking, can induce ciliopathies that may also contribute to the observed decline in smell with ageing [8].

Genetics plays a role in olfaction, as shown by heritability studies that have found low to moderate heritability of odour identification [10,11,12]. Historically, few genetic association studies have examined olfactory identification; more recently, genome-wide association studies (GWAS) have identified SNPs related to olfactory identification in older individuals. In the most recent and largest GWAS to date, Dong et al. [13,14] found nine genome-wide significant SNPs across nine loci for African-Americans (*n* = 1979, mean age = 76.13), but only two SNPs on chromosome 15 and chromosome 10 for Caucasians (*n* = 6582, mean age = 78.83). None of these SNPs overlapped between the two ethnic subgroups.

To date, few studies have examined variation in specific genes implicated in olfaction. In humans, there are over 400 olfactory receptor (OR) proteins, which are primarily responsible for odour detection [15,16]. The OR gene family encodes 500–600 genes and is the largest in the genome. Odour detection and coding by the OR gene family is combinatorial. A single OR can recognize multiple odorants; conversely, a single type of odorant can stimulate several and discrete types of OR [15,16,17]. 

In this study, we aim to identify genes and SNPs associated with age-related olfactory identification in community-dwelling older adults. We focus on the genes from the olfactory receptor family pathways due to their importance in odour identification. Replication of prior results will also be undertaken. Further, to date no other study has examined the genetics of specific odour identification and have instead examined overall olfactory ability. We hypothesize that different OR-related genes and SNPs are involved in identifying individual odours. Additionally, we examine the associations of polygenic risk scores (PRS) for tobacco smoking, AD, PD, WMH, and hippocampal volume (HV) with olfactory identification to ascertain whether genetic risk for these phenotypes is associated with olfaction.

## 2. Materials and Methods

### 2.1. Participants

This cross-sectional study uses participants from two epidemiological studies, the Sydney and Memory and Aging Study (Sydney MAS, UNSW HREC 07001) and the Older Australian Twins Study (OATS, UNSW HREC 07001).

At baseline, Sydney MAS was comprised of 1037 community-dwelling older adults, aged 70–90 years (mean age: 78.84, male: 44.2%) who were invited to participate following random selection from the electoral roll of two government areas of Sydney, NSW, Australia. Comprehensive data were collected including demographic and lifestyle measures, and self-reported medical history. Fasting blood or saliva samples were collected for extraction of DNA and genetic testing. Inclusion criteria included satisfactory proficiency in written and spoken English to complete a psychometric assessment as well as ability to provide informed consent to participate. Exclusion criteria included prior diagnosis of dementia, bipolar disorder, schizophrenia, psychotic symptoms and multiple sclerosis. Informed consent was obtained from all participants. Further details can be found in [18].

OATS recruited 623 twins and their siblings aged 65 and over (mean age: 70.78 years, male: 35.1%) from three eastern states of Australia (Queensland, Victoria, New South Wales) via the Australian Twin Registry. Inclusion criteria included the ability to consent, a co-twin who consented to participate and completion of English at a high-school level. Exclusion criteria included inadequate English proficiency to complete a neuropsychological assessment, diagnoses of malignancy, current diagnosis of psychosis and/or reported psychotic symptoms. Informed consent was obtained from all participants. Similar data and samples were collected as for Sydney MAS. Further details can be found in [19].

Informed consent was obtained from all participants. Ethics approval for Sydney MAS was obtained from the Human Resource Ethics Committees of the University New South Wales and the South Eastern Sydney and Illawarra Area Health Service. Ethics approval for OATS was obtained from the Australian Twin Registry, University of New South Wales, University of Melbourne, Queensland Institute of Medical Research and the South Eastern Sydney and Illawarra Area Health Service. 

Participants were excluded from this study if they had a self-reported history of lung and/or nasopharyngeal malignancy and/or nasal surgery. 

For this study, a subset of 881 and 514 individuals were selected from Sydney MAS and OATS respectively with both genetic and olfactory data available. 

### 2.2. Olfaction

The ‘Brief Smell Identification Test’ (BSIT) is a scratch-and-sniff test consisting of 12 odours: cinnamon, turpentine, lemon, smoke, chocolate, rose, paint thinner, banana, pineapple, gasoline, soap, and onion [20,21]. Participants were asked to identify the odours using a four-category-multiple-choice questionnaire. The BSIT is a forced choice test with each participant being instructed to identify an odour thus yielding a total score out of 12. The BSIT was administered in an identical manner for both Sydney MAS and OATS participants. As in previous total BSIT score research, a maximum of 2 missing responses was accepted and each missing response was assigned a partial score of 0.25 [22]. 

### 2.3. Lifestyle and Health Variables 

Self-reported data on smoking status were collected and a dichotomous smoking variable was used to define current smokers versus past and never smokers. Data on respiratory and nasal health were also self-reported: lung and nasopharyngeal malignancy, previous surgical history and chronic history of respiratory symptoms, i.e. shortness of breath, cough, tachypnoea. Non-English speaking background (NESB) was also self-reported.

### 2.4. Selection of Olfactory Receptor Genes and Single Nucleotide Polymorphisms (SNPs)

Olfactory-related genes were extracted using the SenseLab’s Olfactory Receptor Database (ORDB) (https://senselab.med.yale.edu/ordb/, accessed on 1 August 2019) [23]. The gene *GRCh37* coordinates for 967 available genes were obtained using the Biomart tool of the Ensembl genome browser (https://www.ensembl.org/index.html, accessed on 2 August 2019). The list of SNPs within ±5 kb of the gene coordinates were extracted from the imputed data (Sydney MAS and OATS) using PLINK (*n* = 32,282 SNPs). The effective number of SNPs within each gene was estimated [24] as implemented in the R program matSpDlite.R (https://neurogenetics.qimrberghofer.edu.au/matSpDlite/, accessed on 4 September 2019). To estimate the effective number of independent genes, the correlation matrix of all genes was derived using the first principal component based on the SNP data available within each gene. This gene correlation matrix was used to estimate the effective number of genes using the method mentioned above. 

Candidate SNPs (*n* = 36) were also selected for replication according to the prior literature [13,14,25,26,27] and which were available in our dataset. These SNPs are described in Supplementary Table S1.

### 2.5. Genotyping and Calculation of Polygenic Risk Scores (PRS)

DNA was extracted from peripheral blood or saliva samples using standard procedures [18]. *APOE* genotyping was performed as described in [28] and participants were classified as *APOE* ε4 carriers or not. 

Sydney MAS and OATS samples were genotyped using Affymetrix Genome-Wide Human SNP Array 6.0 (Affymetrix, Santa Clara, CA, USA) and the Illumina OmniExpress array (Illumina, San Diego, CA, USA) respectively. In both cohorts, SNP quality control (QC) was performed using the following filtering criteria to omit SNPs; if (i) sample call rate was <95%, (ii) *p*-value for Hardy-Weinberg Equilibrium was <10^−6^, (iii) if minor allele frequency was <0.01 and/or (iv) strand ambiguous (A/T and C/G) calls. Ethnic outliers were removed based on EIGENSTRAT analysis results [29].

After the QC procedures, additional SNPs were imputed on the Michigan imputation server (https://imputationserver.sph.umich.edu, accessed on 28 September 2016) using the Minimac3 imputation protocol [30] with the European Haplotype Reference Consortium reference panel (HRC r1.1 2016). Imputed SNPs with MAF >0.05, imputation quality score >0.6 and call rate >0.95 were retained for further analyses. 

The imputed SNP dosage scores were converted to genotypes for calculation of each of the PRS (smoking, AD, WMH, HV and PD). All the QC steps and pre-processing were done using PLINK software [31] and the PRS were calculated using the software package PRSice version 2 [32]. The data source used for each of the calculated PRS are described in Supplementary Table S2. The PRS were derived at GWAS *p*-value thresholds of *p* < 5 × 10^−5^. 

### 2.6. Statistical Analyses 

Data pre-processing and descriptive statistics of the olfaction data and potential covariates (age, gender, smoking status, *APOE* ε4 carrier status, non-English speaking background [NESB] status) were undertaken using the IBM SPSS 25 software. All the genetic association analyses were undertaken using the R (version 3.5.2) package (R Core Team, 2018, https://www.R-project.org). To maximize the statistical power, Sydney MAS and OATS were combined for the analyses. 

The 0/1 (incorrect/correct) coded individual BSIT items (*n* = 12) and the log transformed BSIT total score were used as dependent variables in the statistical analysis. 

To account for the correlation amongst twin pairs in the OATS sample, a GLMM with the known covariance structure was used. The kinship coefficient, the probability of sharing two random alleles which are identical by descent, was used as a measure of the relationship between pairs of individuals. Kinship with the individual self and monozygotic (MZ) twin pairs is estimated to be 0.5 and for dizygotic twin pairs it is 0.25. The covariance matrix of the entire sample (Sydney MAS and OATS) was generated using these kinship coefficients. 

The GLMM function glmm with appropriate link function (Gaussian or logistic) as implemented in the R package GMMAT [33] was used for testing associations with the covariates, single SNP replication tests and for the PRS. The single SNP and PRS association tests were adjusted for age, sex and the first 5 genetic principal components. 

Within each gene, the SNP level p-values and correlation among the SNPs were used to undertake gene-level association tests using COMBAT (combined association test) as implemented in the R package COMBAT [34]. Manhattan plots of gene-log (*p*-values) against mid-genomic coordinates of the genes were plotted using the R package GWASTools [35]. 

The false discovery rate (FDR) was used to adjust for multiple testing. FDR adjusted p-values were obtained using the Benjamini-Hochberg procedure [36] as implemented in the R function p.adjust.

## 3. Results

### 3.1. Sample Characteristics 

As shown in Table 1, there were 1395 participants in the Sydney MAS and OATS cohorts with genetic and BSIT data (mean age = 75.52, SD = 6.45). Sydney MAS participants were older and had a slightly lower mean total BSIT score of 9.25 (SD = 2.17) compared to OATS participants (mean score of 9.68, SD = 1.73). A smaller proportion of Sydney MAS participants (3.6%) reported current smoking compared to OATS (5.7%). 

### 3.2. BSIT Item Identification 

As shown in Table 2, with the exception of turpentine (item 2), all items were correctly identified by over 60% of the sample. Turpentine had the lowest correct identification rate in both cohorts at 22.0% (Sydney MAS) and 23.3% (OATS). 

### 3.3. Potential Covariates Influencing Olfaction

The association of potential covariates identified from the literature (age, sex, smoking status, *APOE* ε4 carrier status, NESB status) with BSIT items was examined (Supplementary Table S3). Age was significantly associated with all items and total score with the exception of BSIT items 2 and 5. Sex was inversely associated with total BSIT and all items except 1, 2, 5, 8 and 11, with women scoring better. Current smoking was significantly inversely associated with total BSIT score, although relatively inconsistent across individual items. *APOE* ε4 carrier and NESB status were not statistically significant for any BSIT item nor for the total BSIT score. Hence, age, sex and smoking status were used as covariates for genetic analyses, as these were significantly associated with more than one BSIT item.

### 3.4. Genetic Analyses

#### 3.4.1. OR-Related Genes and Single SNP Analysis

**Figure 1:**
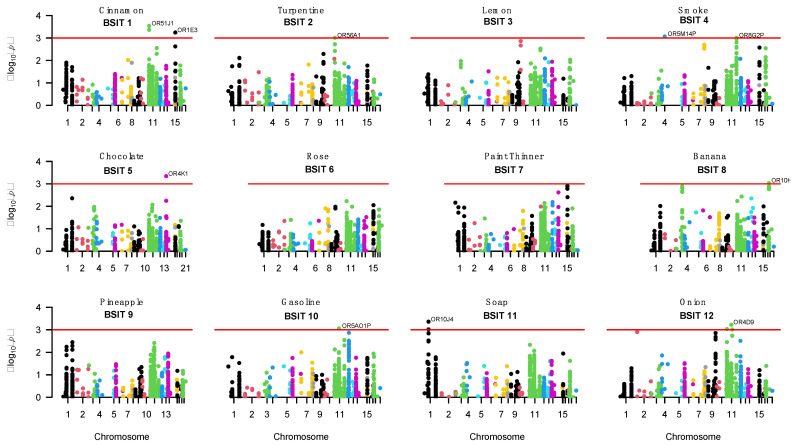
Manhattan plots of the olfactory gene-based association results for all 12 BSIT items. Genes with a *p*-value < 0.001 (red line) are named.

The effective number of independent genes from the OR-related gene family was 447 (out of 967) and there were 9267 (out of 32,282) independent SNPs. Figure 1 shows the Manhattan plots for the results of the gene-based tests with each of the BSIT items. However, none of the gene-based or single SNP association tests was significant for any individual BSIT items or total BSIT scores after correcting for multiple testing. 

Given the number of suggestive gene results and our sample size, we present the top-suggestive hits (*p*-value < 0.001) in Table 3. Suggestive genes for odour identification were found for eight out of the individual twelve BSIT items. Overall, 16 genes were suggestive, with 10 genes mapping to chromosome 11. Cinnamon (BSIT item 1), turpentine (item 2), smoke (item 4), gasoline (item 10) and onion (item 12) were suggestively associated with the genes on chromosome 11. *OR51J1* and *OR51Q1* were suggestively associated with cinnamon identification with both genes belonging to the same family (family 51). *OR8G2P* and *OR8B2* were suggestively associated with smoke identification and were also from the same family. Overall, these results suggest a trend whereby genes associated with a certain scent are found in the same olfactory receptor family. However, in contrast, three genes from chromosome 11 were identified for onion identification (*OR52D1, OR4D9* and *OR7E4P*), which were not from the same OR gene family. 

Six genes were suggestively associated with BSIT items that were not located on chromosome 11 (see Table 3). Interestingly, only three BSIT items did not have any suggestive associations with any chromosome 11 genes: chocolate (item 5) with *OR4K1* (chr 14), banana (item 8) with *OR10H3* (chr 19), and soap (item 11) was suggestively associated with two genes on chromosome 1 from the same olfactory gene family, *OR10J4* and *OR10J1*. 

There were no suggestive results for the BSIT items, lemon (item 3), rose (item 6), paint thinner (item 7) and pineapple (item 9). 

#### 3.4.2. Replication SNP Analysis

Replication analyses were conducted for the identified SNPs from previous studies (*n* = 36) with total BSIT, with no significant results observed. Analyses were also conducted examining individual BSIT items as results for single BSIT items have not been previously reported. Out of the 36 SNPs examined, 13 SNPs were found to be nominally significant (unadjusted *p*-value < 0.05) with individual items (Table 4). 

### 3.5. Polygenic Risk Scores

No significant associations were observed between the various PRS and total BSIT scores. For individual smells, as shown in Table 5, chocolate odour identification (item 5) was significantly associated with the PRS for WMH, smoking and PD (FDR *p*-value = 0.001, *p*-value = 1.15 × 10^−5^, *p*-value = 0.001 respectively). The participants who answered this question incorrectly were at increased genetic risk for WMH, smoking and PD. Cinnamon (item 1) was suggestively associated with the AD PRS, which did not survive multiple testing correction. No other suggestive or significant associations were observed for the PRS for hippocampal volume and AD with any individual BSIT items. All results are reported in Supplementary Table S4.

## 4. Discussion

Where the prior literature has focused on the genetics of global olfactory identification ability via examining total BSIT score, our study of older adults took a novel approach by additionally looking at individual odour identification. Due to the complex and combinational nature of ORs in the identification of odours, we hypothesized that different genetic variants in the OR gene family would be associated with specific odours. No SNPs or genes were significantly associated with olfactory identification. However, we found suggestive evidence for OR genes associated with 9 out of 12 individual odours tested in the BSIT. Additionally, we nominally replicated 13 candidate SNPs identified in prior studies with individual BSIT odours but not the total score. Lower genetic risk for smoking, WMH and PD was associated with correctly identifying chocolate odour. 

In general, age had a significant negative influence on overall BSIT performance, which is consistent with the previous literature [2,7]. However, we only examined older adults and cannot speculate on any change in olfactory identification ability earlier in life. Overall, we found sex differences for odour identification in Sydney MAS and OATS, which is substantiated by previous literature stating that women tend to outperform men on the BSIT [20]. Current smoking status was negatively associated with total BSIT score, this is in line with the original publication by Doty et al. [21]. However, the findings for smoking were relatively inconsistent across individual items.

Only suggestive gene-based association results were observed. The lead SNPs located in each of the genes were predominantly located outside the coding regions of the gene (intergenic, upstream/downstream) except for 4 intronic variants and a synonymous exonic SNP. No common genes were observed across the different odours. Significantly, no results were observed for the individual odours, lemon, rose, pineapple and paint thinner, which may suggest environmental/epigenetic factors influence their identification. 

Two chromosome 11 genes, *OR51J1* and *OR51Q1* (20kb apart), were nominally associated with cinnamon identification, with the two sentinel SNPs in high linkage disequilibrium. These findings lend support to the theory that highly related ORs located at the same locus detect similar odorant molecules [17]. Another gene, *OR1E3* (chr17) was also suggestively associated with cinnamon.

Smoke was suggestively associated with the pseudogene, *OR8G2P* (chr 11). Indeed, olfactory receptor gene families have a relatively high number of pseudogenes [37]. Interestingly, several SNPs have been described that have reverted a pseudogene into a functional one. Smoke was also nominally associated with the gene *OR8B2* on chromosome 11. Turpentine, gasoline and onion were all suggestively associated with chromosome 11 genes. These results suggest chromosome 11 harbors olfactory genes that are important for identification of a variety of odours; indeed, just less than half (42%) of the OR genes are found on chromosome 11 [38]. On the other hand, the identified genes are in gene-rich regions of chromosome 11 and other non-olfactory genes from these regions may be involved in olfactory identification.

Soap identification was one of the three items not associated with any variants on chromosome 11 and it is intriguing that the variants identified were located in the chromosome 1 genes, *OR10J4* and *OR10J1*, with *OR10J4* being located within the latter gene. These results provide further support that highly related OR genes are involved in similar odour identification [17]. Chocolate identification was suggestively associated with a chromosome 14 gene, *OR4K1,* whilst banana was associated with a chromosome 19 gene (*OR10H3*).

It is surprising that none of the previously identified SNPs were associated with total BSIT score, especially those from recent BSIT/SST GWAS [13,14]. However, our sample size was modest compared to these GWAS (*n* = 1395 current study versus ≥ 6252 Caucasian participants). Only 8 out of the 23 GWAS-related SNPs examined were nominally significant with various individual BSIT items. Jaeger et al. [25] ran a GWAS for detection of a particular odour (cis-3-hexen-1-ol), using a very modest sized sample. Of the 11 GWAS SNPs investigated in the current study, two were nominally associated with individual BSIT items (chocolate, gasoline, smoke). Beta-ionone is an odour added to commercial products to give it a pleasant floral note. One SNP that was previously identified (rs6591536, chr 11, nonsynonymous SNP in *OR5A1*) for beta-ionone sensitivity [25] was nominally associated with pineapple odour. We examined a SNP (rs5020278, chr19, nonsynonymous coding change in *OR7D4*) identified by Keller et al. [27] for perception of androstenone and the related molecule, androstadienone (odorous steroids). In the current study it was nominally significant with two odours, cinnamon and lemon.

Utilizing polygenic risk scores (PRS) allowed us to analyze the relationships between individual and total BSIT scores with genetic risk for phenotypes previously associated with olfaction, AD, smoking, WMH, HV, PD. No significant results were found for total BSIT scores. However, those individuals who were at greater genetic risk for PD, smoking and WMH were less likely to identify a chocolate smell. Increased genetic risk for AD was nominally associated with incorrect identification of cinnamon. These preliminary results require replication but suggest that increased genetic risk for smoking, PD, WMH and AD may be associated with lowered specific olfactory identification ability. Specifically, these results suggest that poor chocolate identification may forecast genetic predisposition to PD and smoking. In addition, utilizing cinnamon identification may prove useful for predicting AD risk. However, these results are tentative and require replication. Furthermore, they may not be applicable to younger adults.

Several shortcomings of the current study should be considered. Our sample size was relatively small. There was also limited variation in BSIT scores given the high frequency of correctly answered questions. Hence, replication in independent and larger samples is essential. We did not examine younger individuals hence our results may not extrapolate to this age group. Since our sample was comprised solely of Caucasian adults, our results may not be applicable to non-Caucasians, particularly given a prior GWAS found more olfactory-related SNPs for African-Americans than for Caucasians [14]. We examined only odour identification and did not examine other aspects of olfaction such as perceived intensity, threshold for identification and pleasantness. The PRS that were calculated rely on the relevant GWAS that may explain only a small proportion of the observed variance of the phenotype (e.g. smoking) and hence may not be a very accurate measure of genetic risk. Although we attempted to exclude nasopharyngeal pathology, other conditions such as rhinitis and sinusitis, may have influenced the results.

## 5. Conclusions

In conclusion, this study in older adults evaluated the associations between SNPs and genes of the OR family and various PRS with identification of 12 unique individual odorants in addition to overall olfaction ability. Our findings highlighted that OR-related genes are suggestively associated with BSIT items cinnamon, turpentine, smoke, chocolate, banana, gasoline, soap and onion. We found nominally significant results for 13 out of 36 candidate SNPs previously associated with olfactory phenotypes. Additionally, we identified evidence of associations between chocolate odour identification and PRS for smoking, WMH and PD. Importantly, replication in larger independent samples is needed to confirm these results. Future studies may consider extending this work to younger age groups and to investigate other odours and other facets of smell, such as odour threshold for detection, thus providing a more holistic analysis of the complex sense of smell. 

## Figures and Tables

**Table 1:** Descriptive statistics for Sydney MAS and OATS participants with both genotyping and BSIT data.

Characteristic	Sydney MAS	OATS	Total
N	881	514	1395
Age at assessment (years), mean ± SD	78.7 ± 4.8	70.7 ± 5.5	75.5 ± 6.5
Men *n*, (%)	393 (44.4)	207 (35.1)	600 (41.0)
Brief Smell identification Test (BSIT) score, mean ± SD	9.3 ± 2.2	9.7 ± 1.7	9.4 ± 2.0
SMOKING STATUS *n*, (%)			
Past/Never Smoked	840 (96.4)	558 (94.3)	1398 (95.5)
Current Smoker	31 (3.6)	34 (5.7)	65 (4.4)

**Table 2:** Number and percentage of correct responses for individual BSIT items for Sydney MAS, OATS and total participants.

BSIT Item	Odour	Sydney MAS *n*, (%)	OATS *n*, (%)	Total *n*, (%)
1	Cinnamon	767, (87.1)	455, (88.6)	1222, (87.6)
2	Turpentine	195, (22.1)	121, (23.5)	316, (22.7)
3	Lemon	540, (61.3)	374, (72.8)	914, (65.5)
4	Smoke	658, (74.7)	413, (80.4)	1071, (76.8)
5	Chocolate	749, (85.0)	381, (74.1)	1130, (81.6)
6	Rose	629, (71.4)	408, (79.4)	1037, (74.3)
7	Paint Thinner	785, (89.1)	478, (93.0)	1263, (90.5)
8	Banana	700, (79.5)	421, (81.9)	1121, (80.4)
9	Pineapple	743, (84.4)	460, (89.5)	1203, (86.2)
10	Gasoline	787, (89.3)	487, (94.8)	1274, (91.3)
11	Soap	800, (90.1)	483, (94.0)	1283, (92.0)
12	Onion	785, (89.1)	488, (95.0)	1273, (91.3)

**Table 3:** Olfactory genes suggestively (p < 0.001) associated with individual odours from the BSIT and the sentinel SNPs from each gene ± 10 kb.

BSIT Item ^1^	Odour	Chromosome	Gene ^2^	Gene Based *p*-Value	Sentinel SNP	Sentinel SNP Base Position ^3^	Sentinel SNP *p*-Value	SNP Function/Position
1	Cinnamon	11	*OR51J1*	2.90 × 10^−4^	rs6578634	5420909	4.03 × 10^−5^	Intronic
		11	*OR51Q1*	4.35 × 10^−4^	rs61894107	5442376	3.24 × 10^−5^	Intronic
		17	*OR1E3*	5.73 × 10^−4^	rs769433	3019790	8.12 × 10^−5^	98 bp upstream variant
2	Turpentine	11	*OR56A1*	9.81 × 10^−4^	rs117555183	6049351	5.32 × 10^−5^	380 bp upstream variant
		4	*OR5M14P*	8.59 × 10^−4^	rs1985012	41719745	1.09 × 10^−4^	Intergenic
4	Smoke	11	*OR8G2P*	9.92 × 10^−4^	rs10893174	124090872	5.38 × 10^−4^	Intergenic
		11	*OR8B2*	9.99 × 10^−4^	rs530704	124247361	1.57 × 10^−4^	Exonic (synonymous)
5	Chocolate	14	*OR4K1*	4.57 × 10^−4^	rs9323231	20408911	1.99 × 10^−3^	Intergenic
8	Banana	19	*OR10H3*	9.22 × 10^−4^	rs79876008	15851350	1.38 × 10^−4^	853 bp upstream variant
10	Gasoline	11	*OR5AO1P*	8.74 × 10^−4^	rs11228886	56815339	4.86 × 10^−5^	Intergenic
11	Soap	1	*OR10J4*	4.41 × 10^−4^	rs78689883	159406445	1.99 × 10^−4^	Intergenic
		1	*OR10J1*	9.66 × 10^−4^	rs4128726	159405844	2.08 × 10^−4^	Intronic
12	Onion	11	*OR52D1*	9.30 × 10^−4^	rs4638331	5511431	6.81 × 10^−5^	Intronic
		11	*OR4D9*	6.01 × 10^−4^	rs116937381	59281930	5.72 × 10^−5^	456 bp upstream variant
		11	*OR7E4P*	9.80 × 10^−4^	rs61128173	71334302	3.74 × 10^−5^	Intergenic

^1^ BSIT items 3, 6, 7, 9 are excluded from this table as no genes were associated with these items at *p* < 0.001. Gene-based association tests were performed using combined association test adjusting for age, sex and the first five genetic PCs. ^2^ *OR51J1*: Olfactory Receptor Family 51 Subfamily J Member 1; *OR51Q1*: Olfactory Receptor Family 51 Subfamily Q Member 1; *OR1E3*: Olfactory Receptor Family 1 Subfamily E Member 3; *OR56A1*: Olfactory Receptor Family 56 Subfamily A Member 1; *OR5M14P*: Olfactory Receptor Family 5 Subfamily M Member 14 Pseudogene; *OR8G2P*: Olfactory Receptor Family 8 Subfamily G Member 2 Pseudogene; *OR8B2*: Olfactory Receptor Family 8 Subfamily B Member 2; *OR4K1*: Olfactory Receptor Family 4 Subfamily K Member 1; *OR10H3*: Olfactory Receptor Family 10 Subfamily H Member 3; *OR5AO1P*: Olfactory Receptor Family 5 Subfamily AO Member 1 Pseudogene; *OR10J4*: Olfactory Receptor Family 10 Subfamily J Member 4; *OR10J1*: Olfactory Receptor Family 10 Subfamily J Member 1; *OR52D1*: Olfactory Receptor Family 52 Subfamily D Member 1; *OR4D9*: Olfactory Receptor Family 4 Subfamily D Member 9; *OR7E4P*: Olfactory Receptor Family 7 Subfamily E Member 4 Pseudogene. ^3^ hg19 co-ordinates are given.

**Table 4:** Thirteen SNPs identified from prior studies were nominally significant (unadjusted *p*-value < 0.05) in the current study with individual BSIT items. Results for total BSIT score are also reported.

Chromosome	SNP ^1^	SNP BP	*β* ± S.E.	*p*-Value	Association with BSIT Variable (*p*-Value < 0.05)
4	rs72679931^a^	162245253	0.045 ± 0.023	5.04 × 10^−2^	BSIT Total
			0.490 ± 0.200	1.41 × 10^−2^	Rose (item 6),
			0.659 ± 0.275	1.65 × 10^−2^	Paint thinner (item 7)
			0.644 ± 0.285	2.35 × 10^−2^	Gasoline (item 10)
8	rs34276508^b^	1328679	0.008 ± 0.028	7.77 × 10^−1^	BSIT Total
			0.550 ± 0.221	1.30 × 10^−2^	Lemon (item 3)
8	rs2730141 ^a^	40282221	0.013 ± 0.012	3.02 × 10^−1^	BSIT Total
			0.375 ± 0.177	3.43 × 10^−2^	Onion (item 12)
9	rs4442206 ^a^	73747369	−0.002 ± 0.010	8.35 × 10^−1^	BSIT Total
			−0.207 ± 0.099	3.64 × 10^−2^	Turpentine (item 2)
9	rs6560178 ^a^	73748538	−0.002 ± 0.010	8.50 × 10^−1^	BSIT Total
			−0.207 ± 0.099	3.55 × 10^−2^	Turpentine (item 2)
9	rs2251885 ^b^	103793544	−0.002 ± 0.010	8.52 × 10^−1^	BSIT Total
			−0.253 ± 0.124	4.05 × 10^−2^	Pineapple (item 9),
			−0.394 ± 0.159	1.29 × 10^−2^	Soap (item 11)
9	rs193020892 ^a^	128840966	0.051 ± 0.039	1.91 × 10^−1^	BSIT Total
			1.083 ± 0.445	1.49 × 10^−2^	Gasoline (item 10)
11	rs7938698 ^c^	19709674	0.025 ± 0.016	1.15 × 10^−1^	BSIT Total
			0.338 ± 0.158	3.22 × 10^−2^	Chocolate (item 5),
			0.590 ± 0.204	3.78 × 10^−3^	Gasoline (item 10
11	rs6591536 ^d^	59211188	0.004 ± 0.011	7.08 × 10^−1^	BSIT Total
			0.359 ± 0.121	2.95 × 10^−3^	Pineapple (item 9
15	rs78633367 ^a^	38816290	0.044 ± 0.037	2.64 × 10^−1^	BSIT Total
			0.866 ± 0.435	4.67 × 10^−2^	Onion (item 12)
16	rs964745 ^c^	82540806	−0.022 ± 0.021	3.11 × 10^−1^	BSIT Total
			−0.541 ± 0.240	2.40 × 10^−2^	Smoke (item 4)
19	rs5020278 ^e^	9325116	−0.001 ± 0.013	9.38 × 10^−1^	BSIT Total
			−0.442 ± 0.164	9.00 × 10^−3^	Cinnamon (item 1)
			0.234 ± 0.106	3.04 × 10^−2^	Lemon (item 3)
20	rs6052484 ^b^	4260610	0.014 ± 0.011	1.96 × 10^−1^	BSIT Total
			0.235 ± 0.109	3.05 × 10^−2^	Turpentine (item 2)

^1^ SNPs identified from the literature are ordered based on their chromosome position (hg19). More details on the selected candidate SNPs are provided in Supplementary Table S1. Analyses were performed using GLMM adjusted for age, sex, first five genetic principal components. *p*-values are unadjusted. SNPs were from ^a^ Dong et al. (2017) [14], ^b^ Dong et al. (2015) [13]; ^c^ Jaeger et al. (2010) [26], ^d^ Jaeger et al., (2013) [25]; ^e^ Keller et al., (2007) [27].

**Table 5:** FDR significant and suggestive (*p*-value < 0.05) associations observed between polygenic risk scores for Alzheimer’s Disease (AD), white matter hyperintensities (WMH), smoking, Parkinson’s Disease (PD), and individual BSIT items.

BSIT Odour (Item)	Polygenic Risk Score (PRS)	*β* Value (PRS)	S.E. (PRS)	*p* Value (PRS)	FDR
Cinnamon (1)	AD PRS	0.234	0.080	3.31 × 10^−3^	0.982
Chocolate (5)	WMH PRS	−0.352	0.081	1.70 × 10^−5^	0.001
Chocolate (5)	Smoking PRS	−0.451	0.084	8.87 × 10^−8^	1.15 × 10^−5^
Chocolate (5)	PD PRS	−0.338	0.081	2.67 × 10^−5^	0.001

PRS were based on a GWAS threshold of *p* < 5 × 10^−5^ Full set of results can be found in supplementary data Table S4. Analyses were performed using GLMM adjusted for age and sex. FDR: false discovery rate.

## Data Availability

Data supporting this study is available upon request with the corresponding author, Karen A. Mather. The data are not publicly available due to ethical considerations.

## Supplementary Materials

The following are available online at https://www.mdpi.com/article/10.3390/genes12050669/s1, Table S1. Candidate SNPs previously associated with olfaction, Table S2. Data sources used for calculation of PRS scores, Table S3. Associations between potential covariates and individual BSIT items and total BSIT score, Table S4. Associations between polygenic risk scores based on GWAS threshold <5 × 10^−5^, for Alzheimer’s disease (AD), white matter hyperintensities (WMH), smoking, Parkinson’s Disease (PD), hippocampal volume (HV) and individual BSIT total scores.
