# Supplementary material for: Investigating Olfactory Gene Variation and Odour Identification in Older Adults
Source: Genes (Basel). 2021 Apr 29;12(5):669. doi: 10.3390/genes12050669 (PMC8145954; doi:10.3390/genes12050669)
Supplement: Supplementary file 1 [file genes-12-00669-s001.zip › genes-1185506-supplementary.pdf]

Supplementary Materials:

Supplementary Table S1. *Candidate SNPs previously associated with olfaction*

Chromosome	Candidate Gene	SNP [1]	Olfactory Test Administered	Author, (year)
**1**	*N/A*	rs75904654	BSIT	Dong et al. (2017)
**2**	*OR2J3*	rs4674229	C3HEX identification and threshold test	Jaeger et al. (2010)<br>McRae et al. (2012)
**4**	*N/A*	rs72679931	BSIT	Dong et al. (2017)
**4**	*N/A*	rs41524648	C3HEX	Jaeger et al. (2010)
**5**	*N/A*	rs4865875	BSIT +SST	Dong et al. (2015)
**6**	*N/A*	rs9295791	C3HEX	Jaeger et al. (2010)
**6**	*N/A*	rs4715057	BSIT +SST	Dong et al. (2015)
**6**	*N/A*	rs9321099	BSIT +SST	Dong et al. (2015)
**8**	*DLGAP2*	rs34276508	BSIT +SST	Dong et al. (2015)
**8**	*N/A*	rs1478043	C3HEX	Jaeger et al. (2010)
**8**	*N/A*	rs7000385	C3HEX	Jaeger et al. (2010)
**8**	*N/A*	rs2730141	BSIT	Dong et al. (2017)
**9**	*N/A*	rs16932288	C3HEX	Jaeger et al. (2010)
**9**	*N/A*	rs4442206	BSIT	Dong et al. (2017)
**9**	*N/A*	rs6560178	BSIT	Dong et al. (2017)
**9**	*N/A*	rs16936969	C3HEX	Jaeger et al. (2010)
**9**	*N/A*	rs2245691	BSIT +SST	Dong et al. (2015)
**9**	*PLPPR1*	rs2251885	BSIT +SST	Dong et al. (2015)
**9**	*N/A*	rs193020892	BSIT	Dong et al. (2017)
**10**	*N/A*	rs11200795	C3HEX	Jaeger et al. (2010)
**11**	*N/A*	rs7938698	C3HEX	Jaeger et al. (2010)
**11**	*OR5A1*	rs6591536	β-ionone sensitivity test	Jaeger et al. (2013)
**11**	*N/A*	rs605843	SST	Dong et al. (2015)
**12**	*N/A*	rs1313543	BSIT	Dong et al. (2017)
**12**	*N/A*	rs1313545	BSIT	Dong et al. (2017)
**12**	*N/A*	rs12229599	BSIT +SST	Dong et al. (2015)
**12**	*N/A*	rs3751196	BSIT +SST	Dong et al. (2015)
**13**	*N/A*	rs17252438	BSIT +SST	Dong et al. (2015)
**15**	*N/A*	rs78633367	BSIT	Dong et al. (2017)
**16**	*N/A*	rs964745	C3HEX	Jaeger et al. (2010)
**16**	*N/A*	rs6564086	BSIT	Dong et al. (2017)
**17**	*N/A*	rs199443	BSIT +SST	Dong et al. (2015)
**18**	*N/A*	rs16951602	BSIT	Dong et al. (2017)
**19**	*OR7D4*	rs5020278	Androstenone & androstadienone threshold test	Keller et al. (2007)
**20**	*N/A*	rs6052484	BSIT +SST	Dong et al. (2015)
**20**	*N/A*	rs362582	C3HEX	Jaeger et al. (2010)

Notes: C3HEX:cis-3-hexen-1-ol identification and threshold test , SST: 12 item Sniffin' Sticks screening identification test, BSIT:12 odorant Brief Smell Identification Test.

Supplementary Table S2. *Data sources used for calculation of PRS scores*

Polygenic Risk Score	Data Source	Reference
Smoking	UK Biobank (1239.ukbb.sumstats.gz)	http://ldsc.broadinstitute.org/gwashare/).
Alzheimer's Disease (AD)	https://www.ebi.ac.uk/gwas/downloads/summary-statistics	Kunkle et al. Genetic meta-analysis of diagnosed Alzheimer's disease identifies new risk loci and implicates Aβ, tau, immunity and lipid processing. Nat Genetics. 2019. 51:414-430. doi:10.1038/s41588-019-0358-2
Hippocampal Volume (HV)	ENIGMA and CHARGE Consortia	Hibar, D. Stein, J.L., et al. Common genetic variants influence human subcortical structures. Nature. 2015. 520:224-9. doi: 10.1038/nature14101
Parkinson's Disease (PD)	https://www.ebi.ac.uk/gwas/downloads/summary-statistics	Blauwendraat C, Heilbron K, Vallerga CL, et al. Parkinson's disease age at onset genome-wide association study: Defining heritability, genetic loci, and α-synuclein mechanisms. Mov Disord. 2019;34(6):866-875. doi:10.1002/mds.27659
White Matter Hyperintensities (WMH)	https://www.ebi.ac.uk/gwas/downloads/summary-statistics	Traylor M, Tozer DJ, Croall ID, et al. Genetic variation in PLEKHG1 is associated with white matter hyperintensities (n = 11,226) [published correction appears in Neurology. 2019 Sep 24;93(13):608]. Neurology. 2019;92(8):e749-e757. doi:10.1212/WNL.0000000000006952

Supplementary Table S3. *Associations between potential covariates and individual BSIT items and total BSIT score*

**BSIT item**	**Odour**	**Age**			**Sex**			**Smoking status**			***APOE ε4* carrier status**			**NESB status**		
		β	S.E.	pval	β	S.E.	pval	β	S.E.	pval	β	S.E.	pval	β	S.E.	pval
1	Cinnamon	-0.04	0.01	3.69E-03	-0.25	0.16	1.25E-01	-0.17	0.40	6.76E-01	-0.45	0.18	1.15E-02	0.25	0.28	3.66E-01
2	Turpentine	-0.02	0.01	7.71E-02	-0.02	0.13	8.86E-01	-1.30	0.48	6.32E-03	0.01	0.15	9.54E-01	-0.10	0.22	6.57E-01
3	Lemon	-0.04	0.01	2.42E-05	-0.42	0.12	3.43E-04	0.01	0.29	9.63E-01	-0.22	0.13	9.14E-02	0.25	0.19	1.89E-01
4	Smoke	-0.07	0.01	2.72E-10	-0.62	0.13	3.50E-06	-1.43	0.28	3.42E-07	-0.16	0.15	3.07E-01	0.28	0.22	2.02E-01
5	Chocolate	0.00	0.01	8.68E-01	0.08	0.15	5.89E-01	-0.57	0.31	6.47E-02	-0.25	0.16	1.34E-01	0.12	0.25	6.38E-01
6	Rose	-0.06	0.01	1.90E-08	-0.92	0.13	8.34E-13	-0.20	0.32	5.35E-01	-0.20	0.15	1.74E-01	-0.14	0.20	4.89E-01
7	Paint Thinner	-0.08	0.02	3.46E-07	-1.12	0.20	2.65E-08	-0.74	0.42	7.60E-02	0.27	0.24	2.60E-01	0.31	0.31	3.22E-01
8	Banana	-0.05	0.01	3.94E-05	-0.19	0.14	1.85E-01	-0.29	0.33	3.82E-01	0.24	0.17	1.57E-01	-0.29	0.21	1.62E-01
9	Pineapple	-0.06	0.01	1.37E-05	-0.55	0.16	6.16E-04	-0.02	0.42	9.58E-01	-0.08	0.19	6.60E-01	-0.34	0.23	1.37E-01
10	Gasoline	-0.10	0.02	2.14E-09	-1.04	0.21	9.33E-07	-0.95	0.42	2.28E-02	0.05	0.24	8.52E-01	0.16	0.31	6.19E-01
11	Soap	-0.06	0.02	1.63E-04	-0.51	0.20	1.22E-02	-0.64	0.43	1.32E-01	-0.61	0.21	4.41E-03	-0.11	0.30	7.19E-01
12	Onion	-0.12	0.02	2.77E-11	-0.96	0.21	3.85E-06	-1.25	0.39	1.51E-03	-0.05	0.24	8.23E-01	0.67	0.36	5.99E-02
BSIT Total Score		-0.01	0.00	1.13E-14	-0.09	0.01	2.64E-11	-0.11	0.03	4.68E-04	-0.02	0.02	1.97E-01	0.01	0.02	6.36E-01

Notes: Analyses were performed using GLMM adjusted for age and sex (coded as 0 ([female] and 1 [male]). β: Beta coefficient; S.E.: standard error, pval: p-value.

genes

Supplementary Table S4. *Associations between polygenic risk scores based on GWAS threshold <5x10^{-5}, for Alzheimer's disease (AD), white matter hyperintensities (WMH), smoking, Parkinson's Disease (PD), Hippocampal volume (HV) and individual BSIT total scores*

BSIT Item #	Polygenic Risk Score (PRS)	β value for PRS	S.E. for PRS	P Value for PRS	FDR
1	AD PRS	0.234	0.080	3.31E-03	0.982
1	WMH PRS	-0.071	0.094	4.81E-01	0.982
1	Smoking PRS	-0.112	0.095	2.27E-01	0.982
1	PD PRS	-0.009	0.086	9.16E-01	0.982
1	HV PRS	0.035	0.083	6.77E-01	0.982
2	AD PRS	-0.052	0.067	4.37E-01	0.982
2	WMH PRS	-0.007	0.076	9.24E-01	0.982
2	Smoking PRS	0.0003	0.078	9.96E-01	0.997
2	PD PRS	-0.007	0.074	9.24E-01	0.982
2	HV PRS	0.049	0.066	4.57E-01	0.982
3	AD PRS	-0.823	0.058	1.57E-01	0.982
3	WMH PRS	0.068	0.068	3.14E-01	0.982
3	Smoking PRS	-0.021	0.061	7.30E-01	0.982
3	PD PRS	0.044	0.067	5.02E-01	0.982
3	HV PRS	0.055	0.059	3.45E-01	0.982
4	AD PRS	-0.538	0.066	4.17E-01	0.982
4	WMH PRS	-0.093	0.076	2.21E-01	0.982
4	Smoking PRS	-0.004	0.078	9.63E-01	0.985
4	PD PRS	-0.019	0.074	8.00E-01	0.982
4	HV PRS	-0.023	0.067	7.27E-01	0.982
5	AD PRS	-0.056	0.072	4.38E-01	0.982
5	WMH PRS	-0.352	0.081	1.70E-05	0.001*
5	Smoking PRS	-0.451	0.084	8.87E-08	1.15E-05 *
5	PD PRS	-0.338	0.081	2.67E-05	0.001*
5	HV PRS	0.052	0.072	4.71E-01	0.982
6	AD PRS	-0.012	0.065	8.61E-01	0.982
6	WMH PRS	-0.066	0.075	3.82E-01	0.982
6	Smoking PRS	0.018	0.076	8.14E-01	0.982
6	PD PRS	-0.046	0.073	5.23E-01	0.982
6	HV PRS	-0.055	0.066	4.06E-01	0.982
7	AD PRS	0.159	0.100	1.13E-01	0.982
7	WMH PRS	-0.155	0.113	1.72E-01	0.982
7	Smoking PRS	-0.014	0.114	9.02E-01	0.982
7	PD PRS	-0.69	0.109	5.28E-01	0.982
7	HV PRS	0.150	0.097	1.23E-01	0.982
8	AD PRS	0.029	0.070	6.76E-01	0.982
8	WMH PRS	-0.016	0.080	8.37E-01	0.982
8	Smoking PRS	-0.078	0.081	3.32E-01	0.982
8	PD PRS	0.002	0.078	7.97E-01	0.982
8	HV PRS	-0.049	0.070	4.88E-01	0.982
9	AD PRS	0.036	0.082	6.60E-01	0.982
9	WMH PRS	0.085	0.095	3.72E-01	0.982
9	Smoking PRS	0.021	0.094	8.19E-01	0.982
9	PD PRS	-0.008	0.091	9.33E-01	0.982
9	HV PRS	-0.123	0.082	1.36E-01	0.982

10	AD PRS	0.086	0.105	4.09E-01	0.982
10	WMH PRS	0.162	0.124	1.90E-01	0.982
10	Smoking PRS	0.095	0.122	4.37E-01	0.982
10	PD PRS	-0.049	0.114	6.67E-01	0.982
10	HV PRS	-0.097	0.103	7.95E-01	0.982
11	AD PRS	0.064	0.106	5.47E-01	0.982
11	WMH PRS	-0.032	0.018	7.92E-01	0.982
11	Smoking PRS	0.126	0.124	3.08E-01	0.982
11	PD PRS	0.067	0.117	5.68E-01	0.982
11	HV PRS	-0.029	0.105	7.82E-01	0.982
12	AD PRS	-0.018	0.104	8.60E-01	0.982
12	WMH PRS	-0.008	0.122	9.48E-01	0.982
12	Smoking PRS	0.093	0.125	4.55E-01	0.982
12	PD PRS	0.061	0.118	6.02E-01	0.982
12	HV PRS	-0.029	0.105	3.08E-01	0.982
BSIT Total	AD PRS	-0.006	0.001	3.29E-01	0.982
BSIT Total	WMH PRS	-0.013	0.008	9.99E-02	0.982
BSIT Total	Smoking PRS	-0.008	0.008	3.29E-01	0.982
BSIT Total	PD PRS	-0.011	0.008	1.85E-01	0.982
BSIT Total	HV PRS	-0.0008	0.007	9.10E-01	0.982

Notes. * FDR $p<.05$

References

Dong, J.; Wyss, A.; Yang, J.; Price, T.R.; Nicolas, A.; Nalls, M.; Tranah, G.; Franceschini, N.; Xu, Z.; Schulte, C.; et al. Genome-Wide Association Analysis of the Sense of Smell in U.S. Older Adults: Identification of Novel Risk Loci in African-Americans and European-Americans. *Molecular Neurobiology* **2017**, *54*, 8021–8032, doi:10.1007/s12035-016-0282-8.

Dong, J.; Yang, J.; Tranah, G.; Franceschini, N.; Parimi, N.; Alkorta-Aranburu, G.; Xu, Z.; Alonso, A.; Cummings, S.R.; Fornage, M.; et al. Genome-Wide Meta-Analysis on the Sense of Smell Among US Older Adults. *Medicine* **2015**, *94*, e1892–e1892, doi:10.1097/MD.0000000000001892.

Jaeger, S.R.; McRae, J.F.; Salzman, Y.; Williams, L.; Newcomb, R.D. A Preliminary Investigation into a Genetic Basis for Cis-3-Hexen-1-Ol Odour Perception: A Genome-Wide Association Approach. *Food Quality and Preference* **2010**, *21*, 121–131, doi:10.1016/j.foodqual.2009.08.011.

Keller, A.; Zhuang, H.; Chi, Q.; Vosshall, L.B.; Matsunami, H. Genetic Variation in a Human Odorant Receptor Alters Odour Perception. Nature 2007, 449, 468.

McRae, J.F.; Mainland, J.D.; Jaeger, S.R.; Adipietro, K.A.; Matsunami, H.; Newcomb, R.D. Genetic Variation in the Odorant Receptor OR2J3 Is Associated with the Ability to Detect the "Grassy" Smelling Odor, Cis-3-Hexen-1-Ol. *Chemical senses* **2012**, *37*, 585–593, doi:10.1093/chemse/bjs049.
